# MicroRNA Cues from Nature: A Roadmap to Decipher and Combat Challenges in Human Health and Disease?

**DOI:** 10.3390/cells10123374

**Published:** 2021-11-30

**Authors:** Gurjit Singh, Kenneth B. Storey

**Affiliations:** Department of Biology, Carleton University, Ottawa, ON K1S 5B6, Canada; gurjitsingh@cmail.carleton.ca

**Keywords:** hibernation, anoxia and hypoxia tolerance, freeze tolerance, Oxymirs, mitoMirs, CryomiRs, cancer, ageing, mitochondrial dysfunction, hypothermia

## Abstract

MicroRNAs are small non-coding RNA (18–24 nt long) that fine-tune gene expression at the post-transcriptional level. With the advent of “multi-omics” analysis and sequencing approaches, they have now been implicated in every facet of basic molecular networks, including metabolism, homeostasis, and cell survival to aid cellular machinery in adapting to changing environmental cues. Many animals must endure harsh environmental conditions in nature, including cold/freezing temperatures, oxygen limitation (anoxia/hypoxia), and food or water scarcity, often requiring them to revamp their metabolic organization, frequently on a seasonal or life stage basis. MicroRNAs are important regulatory molecules in such processes, just as they are now well-known to be involved in many human responses to stress or disease. The present review outlines the role of miRNAs in natural animal models of environmental stress and adaptation including torpor/hibernation, anoxia/hypoxia tolerance, and freeze tolerance. We also discuss putative medical applications of advances in miRNA biology including organ preservation for transplant, inflammation, ageing, metabolic disorders (e.g., obesity), mitochondrial dysfunction (mitoMirs) as well as specialized miRNA subgroups respective to low temperature (CryomiRs) and low oxygen (OxymiRs). The review also covers differential regulation of conserved and novel miRNAs involved at cell, tissue, and stress specific levels across multiple species and their roles in survival. Ultimately, the species-specific comparison and conserved miRNA responses seen in evolutionarily disparate animal species can help us to understand the complex miRNA network involved in regulating and reorganizing metabolism to achieve diverse outcomes, not just in nature, but in human health and disease.

## 1. Introduction

**Figure 1:**
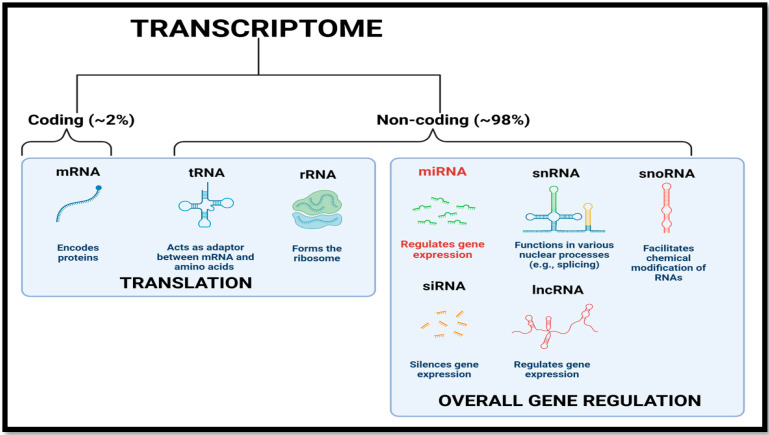
A general representation of the transcriptome depicting various RNA species and their basic functions and characterization. Protein coding mRNAs account for a very small percentage compared with non-coding RNAs involved in overall gene regulation. (Image created with www.BioRender.com, accessed on 26 October 2021)**.**

A primary goal of understanding an animal’s biology is to decipher the data in its genome that provides the information to create and maintain the organism. From worms such as *C. elegans* to humans, the genomes of diverse species contain similar numbers of protein coding genes that actually form barely 2–3% of the transcriptome (Figure 1). Interestingly, the rest of the transcriptome that was long thought to encompass miscellaneous or junk RNA that was not involved in protein coding is now becoming known as one of the most powerful tools for regulating the expression of functional protein coding mRNAs (Figure 1) [1]. These non-coding RNAs include those involved in regulating protein coding machinery (tRNA, rRNA), RNA processing such as small nuclear RNA (snRNA), small nucleolar RNA (snoRNA), microRNA (miRNA) that regulates translation of active coding mRNAs, long chain non-coding RNAs (lncRNA), and short interfering RNAs involved in gene silencing (siRNA) (Figure 1). Of these, miRNAs are categorized as “master regulators” of gene expression and have been shown to regulate about two-thirds of human gene transcripts with effects on all molecular networks including cell survival, senescence and disease [2,3]. The present review focuses specifically on miRNA.

**Figure 2:**
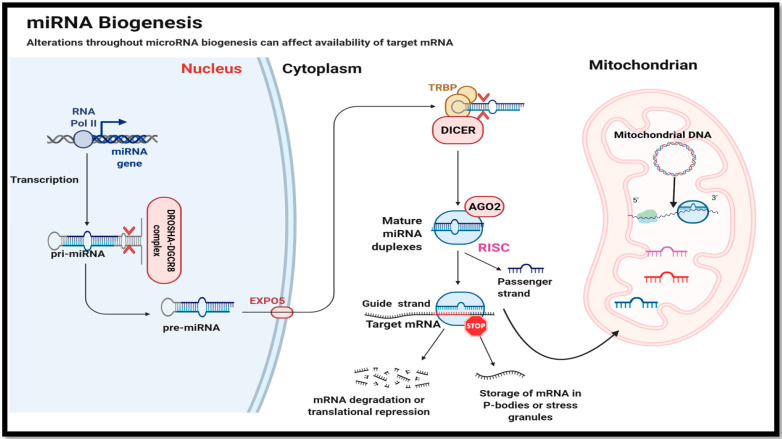
MicroRNA biogenesis. MiRNAs are transcribed in nucleus and pri-miRNA undergoes processing via the DROSHA-DGCR8 complex to form pre-miRNA and transported out to cytoplasm via EXPO 5. Mature miRNAs are generated that facilitate mRNA transcript storage or degradation. MitomiRs can also enter mitochondria to affect expression of mitochondria encoded genes. (Image created with www.BioRender.com, accessed on 26 October 2021).

**Figure 3:**
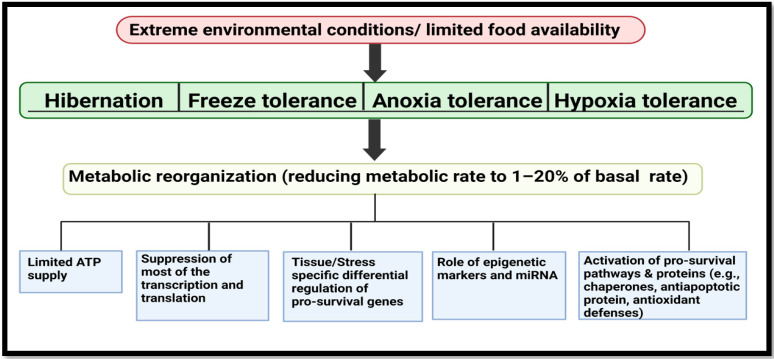
Biochemical and molecular adaptations common to hibernating, freeze tolerant, anoxia tolerant and hypoxia tolerant animals. These animals survive harsh environmental conditions of food and water scarcity and reorganize their metabolic environment (1–20% basal metabolic rate) to survive in the wild. Stress tolerant animals undergo various adaptations (epigenetic modifications, transcription-translation suppression, pro-survival genes activation) with limited ATP supplies to survive under different environmental stresses that can sometimes last up to months. (Image created with www.BioRender.com, accessed on 26 October 2021).

Mature miRNAs are 18–24 nucleotides long and are major players in the reversible regulation of gene expression within cells. They have been explored and characterized in bacteria, viruses, plants and animals [2,4]. The mode of action of miRNA is very well documented; each binds with either full or partial complementarity to the 3′UTR region of a particular set of mRNAs which leads to fates including translation inhibition, degradation, or storage of mRNA transcripts into P-bodies (Figure 2) [5,6]. Small in size and highly conserved across species, miRNAs exert control over major cellular functions since one miRNA species may target many mRNAs or a single mRNA type can be a target of multiple miRNAs by having multiple binding sites in its sequence [5]. This is one of the major reasons why a growing number of reports implicate miRNAs as major players in most of cellular pathways including cell metabolism, stem cell division, cell proliferation, cell cycle control, and cell degradation (apoptosis and autophagy). Furthermore, dysregulation of miRNAs is also associated with disease including diabetes, some cancers and cardiovascular diseases in humans [7,8,9,10]. The pleiotropic nature of miRNAs makes them an effective drug target not only because they interact with protein coding mRNA but also because miRNAs are (i) rapid in action (ii) occur in high copy numbers to regulate mRNA function reversibly, and (iii) are ATP-inexpensive to produce [2]. In order to gain insights and to answer major questions about how specific miRNAs work to regulate genes and processes under cell stress conditions, we need to look no further than to our Mother Nature. Intriguingly, unlike humans, many animal species have developed amazing adaptive abilities to deal with severe environmental stresses, including challenges that deal with food or water restriction, oxygen deprivation (anoxia or hypoxia), or extreme temperature changes. They not only survive but thrive in these challenging environments, often overcoming challenges that would be highly damaging or lethal for humans [2,11,12,13,14]. Interestingly, although the major adaptive strategies used by such stress-tolerant animals are unique from one another and range from hibernation (e.g., thirteen-lined ground squirrels, bears), freeze tolerance (e.g., wood frogs), estivation (e.g., grey mouse lemurs), hypoxia (e.g., naked mole rats, jumbo squids) to anoxia tolerance (e.g., Freshwater turtles), they all undergo a common state of metabolic reorganization [2,15]. This demands regulation at physiological, biochemical and molecular levels to promote (i) a state of metabolic rate depression (MRD) (often to <20% of normal basal rate), (ii) use of limited internal fuel resources (e.g., fat depots, glycogen) to generate ATP sufficient to support prolonged survival and upregulate survival pathways (e.g., anti-apoptosis mechanisms, antioxidant defenses, chaperone proteins, etc.), and (iii) suppression of energy-expensive processes such as protein synthesis and gene transcription (Figure 3) [15]. The present review outlines the role of miRNAs in these natural models of stress tolerance and the potential uses of miRNAs for human applications. We discuss how an understanding of mammalian hibernation has applications to organ preservation and donor transplant, and how low temperature induced miRNAs (CryomiRs) found in cold-hardy organisms or oxygen-sensitive miRNAs (OxymiRs) found in hypoxia/anoxia tolerant species could be applied to human conditions. The review also covers many regulatory networks and signals that influence miRNA action in response to diverse external stimuli to set up a multi-faceted system that coordinates cell/tissue responses to stress across multiple species to implement survival strategies. Ultimately, comparisons of species-specific conserved miRNA responses to stress from a diverse set of evolutionary disparate animals could help us to focus on key miRNA responses in humans. In particular, such studies could set up protocols to curb metabolic rate, allowing for induction of a hypometabolic state, with broad applications ranging from improved management of traumatic injury, disease control, or even development of methods to induce suspended animation for prolonged space travel.

## 2. MicroRNA Biogenesis and Mode of Action

Before discussing the roles of miRNAs in regulation and stress adaptation of cellular pathways, a brief review of miRNA biogenesis and mode of action is pertinent. MiRNA genes are transcribed via RNA polymerase II to produce a primary miRNA (pri-miRNA) transcript (Figure 2) that comes from intergenic regions residing either in an intron or exon of the large non-coding transcript [5]. The pri-miRNA typically ranges from a few hundred to a thousand nucleotides and has a small stem, a terminal loop forming a hairpin and a ssRNA (single stranded RNA) flanking strand [16]. Upon initial transcription and while still in the nucleus, pri-miRNA undergoes enzymatic cleavage by Drosha (an RNAse III endonuclease) that forms a 650 kDa protein complex with DGCR8 (DiGeorge syndrome critical region gene 8) to cleave the pri-miRNA loop near the stem of the hairpin structure. This forms the precursor miRNA (pre-miRNA) (Figure 2) [16,17]. The pre-miRNA is then exported to the cytosol via Exportin-5 (EXPO-5) where another RNAse III endonuclease, called Dicer acts on the terminal loop of the hairpin structure of pre-miRNA to form a mature miRNA duplex (Figure 2). These miRNA duplexes are bound by Argonaute (AGO) proteins and other associated RNA binding proteins such as the transactivation response element RNA-binding protein (TRBP) to form the RNA-induced gene silencing complex (RISC) (Figure 2). To associate the RISC complex with an appropriate mRNA transcript, only a single strand of mature miRNA that is bound by AGO-2 is needed which is called the guide strand [18]. The other miRNA strand (passenger strand) is rapidly degraded and removed (Figure 2). The 5′ region of the guide strand directs the whole RISC complex to mRNA complementarity at the 3′ UTR in a perfect or nearly perfect manner that ultimately leads either to complete degradation of the mRNA or sequestration of mRNA into cytosolic P-bodies or stress granules (resulting in translation suppression) (Figure 2) [5,19]. Nuclear encoded mature miRNAs have also been shown to enter mitochondria where these mitomiRs regulate multiple pathways (Figure 2). Together, these distinct characteristics are what make miRNAs major players in metabolic control and stress adaptation.

## 3. MicroRNA Biology from Extreme Animal Survivalists to Human Health and Disease

### 3.1. Ageing, Oxidative Stress and Related Disorders

Ageing is an inexorable process that results in changes at physiological, biochemical and molecular levels that lead to progressive atrophy, loss of function and/or a diminished capacity to repair or reduce damage products in animal cells, tissues and organs [20,21]. Although progress in this field has helped to enhance the human lifespan, it still remains a challenge to combat ageing-related complications and pathologies including neurodegenerative disorders such as Alzheimer’s disease, cardiovascular problems, and muscle diseases such as muscular dystrophy in older adults worldwide [20]. Numerous microRNAs have been associated with ageing-related molecular pathways such as insulin signaling, mTOR (mammalian target of rapamycin) regulation, reactive oxygen species (ROS) generation and signaling, and DNA damage. This has generated interest in miRNA use as biomarkers [22]. For example, a study of hibernating little brown bats (*Myotis lucifugus*) showed a role for miRNAs (miR-21, miR-1, miR-29b, miR-23a, miR-181b, miR-15a, miR-20a, miR-128 and miR-206) in control of muscle atrophy [23]. Interestingly, despite many weeks of continuous torpor during the winter, these bats show no sign of muscle atrophy and emerge unscathed after arousal. Upregulation of most of these miRNAs was related to the downregulation of pro-atrophy genes, thereby reducing muscle wasting due to inactivity during the winter (Table 1). By contrast, atrophy is a major problem in older adults. Another study of torpor in little brown bats showed downregulation of miR-21 that can promote expression of its direct target, SMAD family member 7 (SMAD-7) [23]. Once activated, SMAD-7 can drive transcriptional regulation of downstream genes that reduce pro-atrophic effects and promote muscle growth and differentiation [24].

Another small hibernator, found in the South American rainforests of Chile, is the marsupial, monito del monte (*Dromiciops gliroides*), that undergoes both daily torpor and prolonged torpor bouts that can last up to five days when ambient temperatures fall to <12.5 °C. These tiny animals show metabolic reorganization similar to brown bats with metabolic rates in torpor as low as 1–5% of euthermic values and strongly suppress all physiological functions [25,26]. The miRNA response by the skeletal muscle of these animals correlated with the molecular responses involved in avoiding muscle wasting during cold exposure and food deprivation. In a recent study, out of 85 miRNAs identified and tested, 11 of the miRNAs were shown to be differentially regulated: seven were upregulated (dgl-miR-1a-1-5p, dgl-miR-1b-5p, dgl-miR-99b-5p, dgl-miR-139-5p, dgl-miR-181a-3p, dgl-miR-190a-5p, dgl-miR-483-5p) and four were downregulated (dgl-miR-16-3p, dgl-miR-22-5p, dgl-miR-33a-5p, dgl-miR-185-5p). Upregulation of the miR-1 family (dgl-miR-1a-1-5p, dgl-miR-1b-5p) appeared to be similar to the situation in hibernating bats and bioinformatics analysis suggested their role in regulating mTOR pathways and promoting MEF-2 (Myocyte Enhancer Factor-2) signaling that is involved in myogenesis and muscle maintenance [26]. The MEF-2 transcription factor is crucial for preventing muscle wasting as its inhibition can diminish oxidative capacity and reduce the expression of type-1 myosin heavy chain [27]. Interestingly, skeletal muscle is involved in thermoregulation in small mammals to produce heat generated by shivering thermogenesis, mostly during arousal from torpor [28,29]. Hence, mechanisms that minimize muscle atrophy are crucial for survival of small mammals in the cold and the role of miRNAs (miR-1 family) is implicated in regulating and activating specific genes, such as MEF-2, to reverse any muscle wasting/damage upon arousal from torpor.

Another example of a mammal that must minimize skeletal muscle atrophy is the biggest hibernating mammal in North America, the brown bear (*Ursus arctos*). Unlike small mammal hibernators (above), their high body mass and thick fur make it impossible for them to reduce their body temperature by more than a few degrees, but their metabolic responses are similar to those of small hibernators (Figure 3). Bears undergo long periods of inactivity (five to seven months) and yet are not affected by muscle disuse atrophy during this time. A recent study also showed MEF2A regulated expression of skeletal muscle miRNA, “myoMiRs” (miR-1, miR-31, miR-23a, miR-29b miR-206) and correlated it with observed increases in transcript levels of *mef2a* that suggest a role of MEF-2 signaling in myogenesis and muscle maintenance during hibernation [30]. Overall, an organized response between muscle growth regulators and pro-atrophy related miRNAs suggests a conserved regulation of key molecular pathways across hibernators despite their body size or habitat in order to promote torpor, thermoregulation and muscle maintenance (Table 1).

Another hallmark of ageing in older adults is oxidative stress, which refers to “the imbalance between the amount of oxidants produced to anti-oxidants to combat molecular damage inside the cell” [31,32,33]. Whereas traditionally used biomarkers and the use of antioxidant therapy have shown therapeutic potential, the use of antioxidant therapies in actual clinical trials have shown null effects in terms of treating disease status and its consequences [34]. However, recent advances in understanding the pathologies of oxidative stress associated with ageing have deemed microRNAs as potential targets or biomarkers of interest which can enhance our understanding on current antioxidant therapies [35,36]. For example, transcription factor, NRF-2 (Nuclear factor erythroid 2-related factor 2), that sits at the top of cellular redox homeostasis and is a key player in inducing many antioxidant genes, is also shown to associate with different miRNAs during oxidative stress [37,38]. The consequences of this oxidative stress could be a decline in mitochondrial function and an increase in apoptosis in cardiomyocytes [39]. Interestingly, a study done with small hibernators, thirteen-lined ground squirrels (*Ictidomys tridecemlineatus*) showed reduced levels of miR-200a affecting *Nrf2* transcript levels in hearts during hibernation [38]. Interestingly, these small mammals that reorganize their metabolic needs to curb ATP use (Figure 3), maintain a high rate of aerobic metabolism; they are bound to face most of the consequences of oxidative stress during arousal from torpor. Interestingly, torpor or a short period of low physiological activities in these animals has been linked with a reduction in cell senescence and ageing in several studies [40,41,42].

Another important organ that impacts human ageing is the brain. As we age, the brain starts to show signs of dysregulated bioenergetics, neuroplasticity and impairment due to oxidatively damaged proteins that can lead to severe neurodegenerative diseases or a stroke [43,44]. Unlike us, the brain of hibernators can repair itself from “tauopathies”. These are pathological conditions under which tau protein is subject to oxidative damage in brain and can undergo aberrant modification via phosphorylation leading to the severe consequences mentioned above [45,46]. Interestingly, during torpor hibernators also exhibit “tauopathies” like those that can cause ageing-related neurodegenerative diseases such as Alzheimer’s disease in humans. However, unlike humans, these tauopathies that develop during torpor are reversible [45]. For example a study done on brain of hibernating bats (*Myotis lucifugus* and *Myotis ricketti*) showed changes in expression of miR-29b that relates with neuroprotection during torpor [47,48,49]. The study on brain of *Myotis ricketti*, using high throughput sequencing, identified 77 bat specific novel miRNAs, out of which 33 were differentially regulated during torpor and were identified as involved in neuroprotective and DNA damage repair functions [49]. The role and the upregulation of miR-153 during torpor was shown to relate with activation of mTOR signaling and inhibition of cell death via KEGG and GO functional predictions [49].

### 3.2. MiRNA: Cancer, Inflammation and Other Diseases

Over the past decade more than 90,000 articles have been published on miRNA regulation in different tissue, cell and animal models. Out of these, around 40–50% of articles specifically focus on altered expression of miRNAs, unique miRNA signaling pathways, or their dysregulation in human diseases including cancer, diabetes, or inflammation. The role of miRNA in cancer has been inducted with a focus on OncomiRs or onco-immunosuppressor miRs in many studies [50,51,52]. There is also great interest in how different cancer types can alter miRNA processing, misexpression and function with a view to searching for new non-invasive biomarkers [53,54]. With scientific advancement and more information on cancer genomes, microRNAs might potentially be used to independently predict the current stage of an active cancer in a patient or as a post-treatment marker, since some cancer models, such as prostate cancer, can recur after surgery [55,56]. For example, a study done on the effects of chemotherapeutic drug treatment on a lung cancer model suggested that a mutation of the P53 transcription factor (the master regulator of and suppressor cell growth) can make drug treatments ineffective by circumventing miR-128-2 induced apoptosis, and thereby promoting resistance to treatments with drugs such as cisplatin [57]. The same study also showed that miR-128-2 targets proteins such as E2F5 to reduce its expression and promote induction of the anti-apoptotic protein p21^waf1^ that is further regulated by p-53 [57]. Interestingly, another study showed that overexpression of miR-205 that induced apoptosis, cell cycle arrest, also inhibited the fanning out abilities of prostate cancer cells to other parts of the body [58]. Other studies emphasizing roles of miRNA have exploited the proteins of miRNA biogenesis which are mostly dysregulated in cancer. For example, many studies have mentioned the role of Drosha proteins in either upregulating or downregulating miRNA expression in a tissue specific manner that can affect the advancement of cancer stages [59]. Another example of a miRNA biogenesis protein is DGCR8, the expression of which is upregulated in different cancers including ovarian and prostate cancers and has been linked with altered miRNA expression and poor prognosis [60]. Ultimately, all these studies have paved the way for miRNAs to be potential biomarkers in the treatment and detection of specific disease symptoms. This is also a requirement for developing precision medicine in the future to provide better prognosis methods and, thereby, better patient survival rates. For example, ThyGeNEXT+ThyraMIR are multiplatform testing kits that exploit the roles of mutational markers (e.g., PTEN, PPARg) as well as miRNA markers (miR-29b-1-5p, miR-31-5p, miR-138-1-3p, miR-139-5p, miR-146b-5p, miR-155, miR-204-5p, miR-222-3p, miR-375, miR-551b-3p) via NextGen sequencing to analyze the pathogenesis of thyroid cancer [61].

Interestingly, despite the progress in making more robust miRNA-based tools, there have been huge challenges in using either human cells or laboratory animals as models. Although experiments have shed light on numerous mechanisms besides miRNA regulation and action, this doesn’t give a complete picture of what is happening in a whole animal. For example, cell-based research does not take into account the importance of cross talk among different cell types in different compartments, as is needed to comprehend physiological functions at molecular, cell, organ or organism levels [62]. On the other hand, traditional animal models in cancer research such as inbred mice are short-lived, genetically homogenous, sometimes immunosuppressed, never exposed to natural habitat, and are often artificially injected with chemicals/cells to induce primary malignancies [63,64]. By contrast, non-traditional models can be excellent models for research in cancer and to study other metabolic disorders. For example, naked mole rats (*Heterocephalus glaber*) that are eusocial and live in underground colonies are known to be one of the best models to study the effects of low oxygen on mammals. Interestingly, the miRNA involvement in responses to hypoxia have been studied and many oxygen-responsive miRNAs (OxyMiRs) on major metabolic pathways were identified. Hypoxia is also one primary cause of tumor induction in some forms of the cancers [65,66]. An international collaborative study from our lab explored the hypoxia-induced miRNA responses in the brain of naked mole rats and showed that many differentially expressed miRNAs were involved in neuroprotection and anti-apoptotic responses [67]. For example, miR-24 that was upregulated in the brain is known to be involved in hypoxia-induced reduction of cytochrome c, a mitochondrial protein involved in inducing apoptosis [67]. Another study showed that downregulation of miR-335 that regulates the hypoxia-inducible transcription factor-1 (HIF-1) in brains of hypoxic naked mole rats can activate genes involved in glycolysis under low oxygen availability [68,69]. Furthermore, the study showed an upregulation of miR-155 in mole rat brain that targets HIF-1α and also induce the expression of NF-κB transcription factor that is involved in antioxidant defense, DNA damage repair, and the inflammatory response. NF-κB induction can maintain miR-155 levels during hypoxic stress suggesting stringent regulation at a posttranscriptional level by players in the cell stress response machinery [69,70]. A study of hibernating grey mouse lemurs (*Microcebus murinus*) from Madagascar also showed miRNA action in regulating genes involved in cellular defense systems. For example, increased expression of miR-92a, miR-193b and miR-218 relates to their role in regulating P53 signaling, the mTOR pathway and inhibits key protein synthesis, respectively, during torpor in lemurs [71,72,73,74]. Interestingly, like bears, these lemurs are also warm hibernators and follow the common theme of undergoing metabolic reorganization and activating downstream responses to enter daily or multi-day torpor as needed.

Besides their role in regulating pro-survival pathways, miRNAs also regulate multiple cell function individually via influencing the synthesis of key metabolic enzymes in diverse metabolic pathways. For example, the role of fatty acid synthase (FAS) in lipid metabolism has made it a promising clinical target for use as a biomarker for FAS-positive tumors and benefits patients with therapies that include treating with FAS inhibitors such as TVB-2640 [75,76]. However, a complete understanding of the regulation of the *fasn* gene (that encodes FAS) at multiple levels could further boost the outcome of FAS based therapies. Interestingly, one study of the hibernating ground squirrel showed miR-195 involvement in regulating *fasn* transcripts during torpor. Levels of miR-195 that target the *fasn* transcript are correlated with lower protein levels of FAS protein in liver [77]. This post transcriptional regulation of a key enzyme involved in fatty acid metabolism could be a putative target to explore in hepatic cancer models.

Other studies from our lab specifically focussed on OxyMiRs that are expressed under conditions of low or no oxygen and are involved in overall metabolic regulation [78]. These showed differential regulation in diverse species and tissues and in response to the different low oxygen stress conditions. Some of these OxyMirs could be relevant to research on cancer or other metabolic diseases [78]. An intriguing example is the jumbo squid (*Dosidicus gigas*), that migrates daily from 300 m depths where hypoxic conditions (~10 µM oxygen) are severe but rises to surface waters every night to feed [79]. Out of a group of 39 miRNA examined, miR-2 family (miR-2a-3p and miR-2c/d-3p) showed increased expression in hearts of hypoxic squid. The miR-2 family has links to suppressing pro-apoptotic genes such as TGF-β whose inhibition has been shown to suppress proliferation and tumor migration [80,81]. The same study also reported a role for miR-133 in hypoxic brain and heart during recovery from ischemic injury and cardiac hypertrophy, respectively [80,82,83].

Among vertebrate, a major model of anoxia tolerance are turtles of the *Trachemys* and *Chrysemys* genera that winter under water in ponds/lakes that often become ice-locked and oxygen-depleted. For example, the red-eared slider (*Trachemys scripta elegans*) can survive for up to four months in oxygen depleted cold water [84] by undergoing metabolic reorganization (Figure 3). This leads to a focus on ATP production by anaerobic glycogenolysis with lactate accumulated as the end product of the Warburg effect in various cancer models. Whereas high lactate levels normally lead to acidic cell conditions, turtles have ways to elude acidosis by storing lactate in their shells [85,86]. Studies of turtles showed a prominent increase in the p-53 transcription factor that led to upregulation of genes (e.g., *GADD45a, PGM, 14-3-3sigma)* that code for proteins involved in arresting cell proliferation, DNA repair and apoptosis to minimize energy usage under anoxia [87]. Mir-34 was also upregulated during anoxic submergence and has a role in promoting cell cycle arrest under control by the p-53 signaling network [87]. Another study assessing anoxia-induced cell cycle arrest in turtles showed that elevated levels of miR-15a and miR-16 can affect cyclin D1 protein levels that are essential for cell cycle initiation and, thereby, suppress cell proliferation under anoxic conditions (Table 1) [88].

Whereas miRNA-based regulation has been shown to regulate transcription factors, such as the p-53 “master regulator” discussed above, studies have also shown that miRNAs also has roles in modulating networks during stress via altering expression of small proteins involved in biochemical “tagging” of large proteins via ubiquitination or SUMOylation [89,90]. Although both of these are posttranslational modifications known to act as crucial players in targeting proteins for proteasomal degradation, studies have also shown their role in cell signaling, protein localization and movement, protein stability and other pro-survival pathways [91,92]. Dysregulation of these processes are detrimental to the activity of proteins and can lead to tumor development [90]. Interestingly, a study done on brains of 13-lined ground squirrels reported a decrease in the miR-200 family (miR-200a,b,c/miR-141/miR-429) and miR-182 family (miR-182/miR-183/miR-96) caused by an increase in global SUMOylation and ubiquitin-like modifiers (ULMs) such as neuronal precursor cell-expressed developmentally down-regulated protein 8 (NEDD8), ubiquitin fold modifier1 (UFM1), and fan ubiquitin like protein-1 (FUB1) that protect cells from ischemic damage under low oxygen and glucose-deprived conditions during torpor [91,93]. Ultimately, this study not only correlated the role of the cell’s degradative pathways with action by the miR-200 and miR-182 families that also targets cancer metabolism in the brain, but also hinted that they might be playing a possible role in glucose metabolism, which is interesting as cancer cells rely on glucose as their primary metabolic fuel [92,93,94].

Another important consideration in understanding the regulation of metabolic disorders such as obesity, hepatic steatosis and cardiovascular diseases is the role of brown adipose tissue (BAT) that can act as a “metabolic hoover” for nutrients so as to promote substrate oxidation and dissipating heat via non-shivering thermogenesis (NST) [95,96,97]. Indeed, brown fat is crucial to regulating whole body homeostasis in many small mammals by releasing signaling molecules such as cytokines that are involved in an immune response. Interestingly, the regulation and function of BAT, along with its role in inflammatory signaling, has been explored in hibernators, such as 13-lined ground squirrels, and could potentially illuminate the underlying biology of this tissue in regulating ROS during arousal from torpor as a result of NST [98]. A recent study using small RNA sequencing reported miRNA expression patterns of 76 differentially regulated miRNAs in BAT in hibernating 13-lined ground squirrels during prolonged torpor [13]. For example, miR-365-5p was highly expressed and shown to target a number of genes involved in regulation of adipogenesis and in modifying miRNA biogenesis such as Ankyrin Repeat Domain 52 (ANKRD52), that dephosphorylate AGO-2 during torpor [13,99,100]. Interestingly, miR-99b-5p showed higher expression during torpor that can target a BAT-secreted protein, FGF-21 (fibroblast growth factor 21). FGF-21 is involved in maintaining the body’s homeostasis via regulating glucose oxidation and lipid catabolism [13,100]. Similarly, miR-92a-3p that was also upregulated during torpor in the same study was suggested to inhibit selected processes in BAT including UCP-1 activity, brown fat differentiation, and oxygen utilization during torpor [13,101]. Contrary to these results, a separate study on ground squirrels identified two downregulated miRNAs during torpor (miR-103 and miR-107) that correlated to a role in enhancing BAT mitochondrial regulation [102]. These miRNAs could play an important role in activating brown fat to increase NST during arousal from torpor. The activated BAT can alter energy metabolism by elevating energy expenditure and inhibiting body fat storage, and might contribute to anti-obesity strategies [102,103].

### 3.3. Mitochondria Specific MiRNA, MitoMirs and MDPs: Dark Horses Involved in Mitochondrial Dysfunction?

Not only are mitochondria the powerhouse of the cell but they have crucial roles in driving cellular machinery and regulating major metabolic and signaling pathways with respect to both internal (organism) and external (environmental) cues. They are at the center of redox signaling with high rates of ROS production that, if uncontrolled, can damage mitochondrial DNA (mtDNA) and impair respiratory chain machinery, membrane permeability, and defense systems, leading to a buildup of damaged proteins [104]. This can ultimately lead to a wide variety of health problems, including strokes, metabolic disorders such as diabetes, unchecked tissue growth and tumor development [104]. As mentioned previously, mitochondria play crucial roles in hibernators, hypoxia/anoxia tolerant, and freeze tolerant animals, particularly in the reduction and reprioritization of metabolic machinery during stress-induced metabolic rate depression. Yet all of these stress-tolerant animals are unharmed when they recover from their respective stress situations. For example, a study done on 13-lined ground squirrels showed liver specific regulation of miR-15b and miR-25 during hibernation [105]. Interestingly, miR-15b has been shown to play a crucial role in inhibiting cell proliferation, ROS production and downregulating mRNA and protein levels of Bcl-2 (B-cell lymphoma 2) that is a sentinel of mitochondrial apoptosis [106]. Upregulated levels of miR-25 coincide with its role in inhibiting PTEN (phosphatase and tensin homolog), that when activated (e.g., via miR-25 knockdown) functions in the PI3K/Akt/PTEN pathway and can induce mitochondrial cell death [107].

Besides being the “end function” organelle in regulating redox signaling and cell death, recent studies have also shown a role for mitochondria-derived peptides (MDPs) that are encoded mainly within the 16S ribosomal RNA gene of mitochondria. The first such peptide was humanin (21–24 amino acids) that has a cytoprotective action [108,109,110]. Since its discovery in 2001, humanin (HN, MTRN2) has been shown to occur widely in mammals and has recently been reported in anoxia-tolerant turtles [110]. Its role appears to be shielding cells from redox damage, chemotherapy and induced side effects, cytotoxic insults, and hypoxia and suggest that humanin might be playing a crucial role in abating effects of mitochondrial dysfunction [108,111,112,113]. Interestingly, humanin is highly conserved across mammals [114] and has also been found computationally in various models of vertebrate stress tolerance which suggests that it might have an ancestral role in cytoprotection, protecting cells from oxidative damage during stress [109,110]. Studies of humanin have explored its role at multiple levels but many aspects of its functions in retrograde signaling still need to be deciphered. Recent studies have outlined a role for miRNAs in regulating expression of this MDP [115,116,117]. The role of miRNA-155 was linked to glucose-induced apoptosis but this was inhibited when treated with humanin [116].

Another interesting offshoot of humanin research is the discovery of mitochondrial miRNA (mitomirs), a subgroup of miRNA derived mostly from nucleus but imported into mitochondria (Figure 2) where they are linked with various of mitochondrial functions ranging from energy metabolism to apoptosis [118]. For example, two mitomirs in humans (hsa-miR-mit3 and hsa-miR-mit4) have been shown to bind to the HN gene [119]. The potential roles of mitomirs in controlling energy metabolism in mitochondria has begun to unravel [120]. MitomiR-181c has been shown to suppress the levels of mitochondrial encoded cytochrome c oxidase 1 (COX-1), a subunit of complex IV of the electron transport chain [120], and is also a target for NSAIDs (non-steroidal anti-inflammatory drugs) [121]. Other studies have shown that mitomirs can act as novel players in cardiovascular diseases and mitochondrial dysfunction. One study showed that levels of mitomiR-696, mitomiR-532 and mitomiR-690 rose during the initial stages of heart failure, linking these to changes in mitochondrial energetics [122]. Another study showed a potential role of mitomiR-378a in regulating glucose homeostasis via inhibiting mitochondrial ATP synthase subunit 6 (MT-ATP6). In addition, mitomiR-378a has been shown to inhibit lactate dehydrogenase and induce apoptosis in cardiomyocytes [123]. Other mitomiRs such as let-7b, miR-146a, miR-19b, miR-34a and miR221 all contribute to cell senescence [124]. From this study, mitomiR-146a was shown to target Interleukin 1 Receptor Associated Kinase 1 (IRAK-1) and TNF receptor-associated factor 6 (TRAF-6) which are mediators of immune response and activate cell senescense associated with interleukin-6 (IL-6) [125,126]. Other miRNAs (miR-19b, miR-34a and miR-221) can regulate Bcl-2 that is a crucial player in antioxidant, antiapoptotic and autophagic responses as explained earlier. Overall, many studies have established the role of mitomiRs in cardiovascular diseases and established their roles in either regulating glucose metabolism, mitochondrial energetics or genes in mitochondrial metabolism suggesting a possible future role for mitomiRs in mitochondrial-based therapeutics [127,128,129].

### 3.4. MicroRNAs in Organ Preservation and Transplantation

Since the time that the first successful organ transplant was completed nearly 70 years ago, it has remained a challenge to fulfill the demand for organs eligible for transplantation [130,131]. This is not only because of low availability of suitable donor organs but also due to lack of robust methods for long term preservation of excised organs [132]. For example, in the USA, 70% of heart donations go unused with less than 10% of eligible candidates receiving timely donations [132]. The method that has been used for decades for preserving organs is static cold storage (SCS), storing organs at 4 °C in an ice cooler and rushing to the recipient before damage to the organ becomes irreparable [133]. The hypothermic conditions reduce cell metabolic rate and provide some cytoprotection. Over time, perfusion cocktails have been developed that further stabilize organs during the transfer from donor to recipient. Some of the advances in cryopreservation have come from mimicking the metabolic adaptations used by naturally freeze-tolerant animals [15]. The SCS method does pose challenges that include (i) combatting with tissue damage due to prolonged cold storage and ischemic conditions, and (ii) having no direct “subjective” method for medical professionals to ascertain the actual condition of an organ before transplant [134].

Hibernation studies from our lab, particularly on marsupials, rodents and primates, have highlighted some of the challenges mentioned above and revealed evolutionary strategies for protecting cells/organs during prolonged torpor (under MRD) including cellular defenses (antioxidant, antiapoptotic, etc.), miRNA responses, suppressing mTOR signaling and other pathways (Figure 3 and Table 1). All these suggest that an ancient “switch” could be hidden in the human genome that could trigger torpor in humans or be induced to extend donor organ preservation times [135,136,137]. Indeed, miRNA plays an important role during the transitions between active vs torpid states in multiple animal models [2,71,138]. For example, in 13-lined ground squirrels, higher expression of miR-193a has been linked to inhibiting key activators of the immune response such as mTOR, that can activate T cell proliferation & signaling to sense immune signals (e.g., cytokines, antigenic signals) [138]. Interestingly, a separate study showed mTOR inhibition in ground squirrels that correlated with the above study in bringing a state of hypometabolism by inhibiting energy expensive processes such as protein translation and also by diminishing immune response during stress [138,139,140]. Another interesting example is a warm hibernator, the gray mouse lemur. Upregulation of miR-874 in liver during torpor can provide an interesting link in regulating toll like receptors (e.g., TL-4) that have shown to activate stress/damage associated downstream responses in transplanted organs and contributes to ischemic/reperfusion injury mediated allograft rejection [71,141]. Another miRNA of note was downregulated during torpor; miR-222 is a promoter of insulin resistance that can affect the long term survival rate of grafts and reduce rejection rates after transplant [71,142]. Interestingly, these findings align with the recent advances in organ preservation that have pushed the research from cold reperfusion to normothermic reperfusion or warm organ preservation [134,143].

### 3.5. Low Temperature MiRNA Target Selection: A Possible Role in Inducing Therapeutic Hypothermia?

Therapeutic hypothermia (TH) is another intriguing area of research. This is a targeted temperature management strategy involving moderate cooling of the body to reduce organ oxygen consumption (particularly by the brain) in order to improve tissue recovery following traumatic injury [144,145,146]. Therapeutic applications of this treatment reduce damage in patients dealing with hypoxic-ischemia insults such as cardiac arrest or stroke and improve neurological recovery after traumatic injuries [144,145]. Over the last 10–15 years there have been multiple studies that underscored the importance of low temperature in targeting genes in different pathomechanisms such as inflammation, apoptosis or DNA fragmentation [147,148,149]. Some of these studies shed light on our understanding of basic molecular and cellular pathways that underlie the beneficial roles of posttraumatic hypothermia, yet many questions still need to be answered, including the roles of posttranscriptional regulation. Both cell cultures and animal model studies have demonstrated the effects of many miRNA species under hypothermic versus normothermic conditions [150,151]. For example, a study of the role of temperature change in dealing with traumatic brain injury (TBI) showed that lower levels of miR-874 under hypothermic conditions could promote cell function by upregulating the levels of essential proteins for normal cell function (e.g., cytosolic or membrane proteins), suggesting role of cooling temperatures in inducing miRNA expression during TBI [150]. The same study also showed an upregulation of miR-497 and miR-290 (post TBI hypothermia), that are involved in regulating protein movement and transport whereas downregulated levels of miR-9 could promote transcription and increase the amount of protein involved with actin binding or the plasma membrane, thereby helping to maintain cell and cytoskeleton integrity [150].

Furthermore, binding between miRNA species and their target mRNAs is also temperature sensitive; binding is stronger as temperature declines. This concept has been explored in ectothermic animals by our lab with computational studies showing that as thermodynamic free energy used to predict miRNA-mRNA stability decreases, miRNA-mRNA target binding increases [152]. For example, target prediction for miR-21 showed that the number of mRNA targets that could be bound by miR-21 rose from 47 to 756 when temperature was lowered from 37 °C to 3 °C, as determined by a computational analysis using miR-21 sequenced from hatchling painted turtles [153]. Another example of this are North American wood frogs, *Rana sylvatica,* that are the best studied model of vertebrate freeze tolerance and have been shown to regulate tissue specific regulation of multiple metabolic pathways by posttranscriptional controls [154]. They are a perfect model to enhance our current understanding of low temperature miRNA regulation and also decipher concepts of cytoprotection for organ preservation. These frogs undergo multiple changes as they freeze, including: (a) up to two-thirds of cell water is drawn out of tissues/organs to join extracellular ice masses, and (b) cells rapidly accumulate a huge amount of cryoprotectant, glucose in the case of wood frogs, that allow cells to retain viability despite a shrunken size and extreme osmolality. At the same time, MRD is used to “pull the plug” on energy-expensive nonessential metabolic processes and support long-term survival in an anoxic frozen state for months until the spring thaw [15,155,156]. Similar to hatchling turtles during freezing, miR-21 levels were also elevated in the liver and muscle of wood frogs during 24 h freezing at −2.5 °C. This miRNA targets mRNAs for inflammatory caspases such as caspase-3, and can promote anti-apoptotic factors such as apoptotic protease activating factor-1 (*apaf-1*) during the freezing episode [157]. Another study of wood frogs brains during freezing showed upregulation of miR-451 that can act to promote protection from anoxia/reperfusion injury [158]. Interestingly, miR-181a-3p was downregulated in the same study (during thawing at 5 °C) which is linked to brain recovery from ischemic conditions via restricting apoptosis and promoting activity of pro-survival proteins. Indeed, targeting such cell processes is a major requirement for the therapeutic hypothermia [159,160]. Another important study highlighted the role of miRNA at low temperatures in wood frog hearts during thawing. The levels of miRNA-145 were high in frozen frogs but decreased during thawing, suggesting their role in reactivating cell systems involved with proper heart function during recovery after thawing that can include reversing ischemia, dealing with reperfusion injuries and with physical damage by ice to tissues [161]. This study is of particular interest since it again shows the involvement of miR-145 in dealing with temperature changes and freezing stress.

A third study on the anoxia-tolerance of the red-eared slider turtle (*T.s. elegans)* also reported changes in miRNA expression levels at low temperatures (5 °C) in liver, white skeletal muscle, kidney and spleen [162]. For example, under 20 h anoxia stress, miR-20a showed elevated expression in all tissues tested. This miRNA can have a crucial role in inhibiting cell proliferation, suggesting that anoxia-specific conserved miRNA regulation was active in all tissues to suppress mRNA targets involved in ATP expensive pathways [162]. Similar to wood frogs, *T.s. elegans* showed elevated levels of miR-21 in kidney, muscle and spleen and this also correlated with *apaf1*, *casp3* and *casp7* regulation, suggesting a similar role in mediating an anti-apoptosis response under anoxia exposure [162,163]. Another study using a hypothermic rat model showed activation of miR-374-5p in skeletal muscle in response to hypothermia (12 °C), and inducing suppression of *Mex3B* (muscle excess 3) that ultimately affects genes involved in the apoptosis cascade [151]. Overall, these studies highlight the roles of temperature and stress specific miRNAs in activating different regulatory pathways and mediating positive metabolic consequences similar to the effects of therapeutic hypothermia after traumatic injury and may provide a guide to comprehend temperature/stress specific miRNA regulation involved in these basic cellular mechanisms.

**Table 1:** List of miRNA studies discussed herein in different stress tolerant animal models as well as other animal models.

miRNA Species	Animal/Cells	Putative Target/Pathways	Reference
miR-21, miR-1, miR-29b, miR-23a, miR-181b, miR-15a, miR-20a, miR-128, miR-206	*Myotis lucifugis*	Muscle atrophy	[23]
miR-29b	*Myotis ricketti*	Neuroprotection	[49]
miR-1 family	*Dromiciops Gliroides*	MEF-2 signaling in myogenesis and muscle maintenance	[26]
miR-1, miR-31, miR-23a, miR-29b, miR-206	*Ursus arctos*	MEF-2 signaling in myogenesis and muscle maintenance	[30]
miR-365, miR-99, miR-92a, miR-103, miR-107	*Ictidomys tridecemlineatus*	Affects adipogenesis, levels of FGF-21 during torpor and BAT mitochondrial regulation respectively	[13,102]
miR-200a, miR-15b, miR-25	*Ictidomys tridecemlineatus*	Antioxidant response (NRF-2), suppress cell proliferation and mitochondrial apoptosis respectively	[38,105]
miR-195	*Ictidomys tridecemlineatus*	Fatty acid synthase regulation	[77]
miR-200b, miR-200c, miR-141, miR-429, miR-182, miR-183, miR-96	*Ictidomys tridecemlineatus*	Targets SUMOlyation and Ubiquitin like identifiers (ULMs)	[89]
miR-24	*Heterocephalus glaber*	Hypoxia induced reduction in mitochondrial protein, cytochrome c	[67]
miR-335, miR-155	*Heterocephalus glaber*	Regulating HIF signaling and NF-ĸB respectively	[69]
miR-92a, miR-193b, miR-218, miR-222, miR-874	*Microcebus murinus*	P53 signaling and cell survival pathway	[71]
miR-2 family, miR-133	*Dosidicus gigas*	Suppressing pro-apoptotic genes and Ischemic injury recovery in cardiomyopathy respectively	[80]
miR-34, miR-15a, miR-16	*T.s elegans*	Suppress Cell cycle and P53 signaling	[87,88]
miR-20a, miR-21	*T.s elegans*	Induce anti-apoptotic response	[162]
miR-21	*Rana sylvatica*	Targets mRNA for inflammatory caspases, casp-3	[157]
miR-451, miR-181a	*Rana sylvatica*	Reduces anoxia/reperfusion injury via restricting apoptosis	[158]
miR-145	*Rana sylvatica*	Reduces ischemic injury	[161]
hsa-mir-mit3, hsa-mir-mit4	*Homo sapiens*	Humanin gene regulation in the mitochondria	[119]
Let-7b, miR-146a, miR-19b, miR-34a, miR-221	*Homo sapiens*	Cell senescence	[124]
miR-128-2, miR-205	*Homo sapiens*	P53 signaling and anti-apoptotic response	[57,58]
miR-874, MIR-497, miR-290	*Rattus norvegicus*	Increasing transport of cytosolic and membrane proteins	[150]
miR-9	*Rattus norvegicus*	Reduced levels promote transcription and translation in generating proteins for cell and cytoskeleton integrity	[150]
miR-274	*Rattus norvegicus*	Inhibit Mex3B and affecting genes in apoptotic cascade	[151]
mitomiR-181c	*Rattus norvegicus*	Decrease COX-1 in ETC chain	[120]
mitomiR-696, -532, -690	*Mus musculus*	Mitochondrial energetics in heart failure	[122]
mitomiR-378a	*Mus musculus*	Suppress mitochondrial ATP6 and induce apoptosis	[123]

## 4. MiRNA Therapeutics and Conclusion

There are nearly 39,000 entries of miRNA sequences for over 250 species on the miRbase database (miRBase 22.1 Release, October 2018), out of which only 5% are human sequences [164]. Although insight into the role and action of different miRNAs in humans can aid in comprehending complex cellular networks and mechanisms underlying diseases [165], the role of miRNAs in many other animal models and their biological relevance to human diseases and diagnosis is what makes miRNAs attractive as novel therapeutic targets. At present, miRNA-based therapeutics are focused mainly on finding potential non-invasive miRNA biomarkers or drug targets for use in diagnosis of human diseases, and finding ways to push them from the research bench to the bedside [166]. Interestingly, both miRNA antagonists (antimirs or antagomirs) and miRNA mimics have shown some promise in clinical trials. For example, in the case of liver cancer, the miRNA mimic MRX34 enhanced the activity of miRNA-34 in phase-1 clinical trials [167]. Another study that has entered clinical trials involves an antisense RNA oligo called LNA miravirsen that has complementarity to the 5′ end of miR-122 and suppresses its action; it has been used for the treatment of the Hepatitis C virus and for the lowering of cholesterol in a non-human primate model [168,169]. Recent research has also paved the way for diagnostic tools and kits (e.g., ThyGeNEXT+ThyraMIR) that support the use of miRNAs as potential non-invasive biomarkers (detected in body fluids after biopsy); for example, in detecting the thyroid cancer phenotype [61]. Despite some development in this field and with several studies registered for miRNA-based clinical trials with the National Institutes of Health (https://clinicaltrials.gov, accessed on 26 November 2021), to make robust miRNA-based drugs, there remain some gaps in understanding regarding the underlying principles of many diseases and how cellular machinery responds to various metabolic challenges. As mentioned in this review, a comparison of species-specific conserved miRNA responses from diverse sets of evolutionary disparate stress tolerant animals can help us to uncover miRNAs responses and effects in humans. The possible outcomes of this could be a better understanding of the underlying principles of MRD and the regulating of allied cellular networks in the field of organ transplant, metabolic disorder research, ageing and therapeutic hypothermia via implicating roles of multiple players that not only include miRNAs but other major players such as transcription factors, metabolic enzymes and cytokines etc., in order to show a complete picture of metabolic regulation of a whole animal at organ/cell/stress specific level [170]. Indeed, such studies could also help to understand the underlying consequences of different cellular networks that might operate in humans but may be dynamically regulated in stress tolerant animal under different stress conditions. Overall, the large number of miRNA species identified in unique animal models and comprising tissue specific (myomiRs), temperature specific (cryomiRs), organelle specific (mitomiRs) miRNAs and stress specific (OxymiRs), and potentially other groupings, could pave the way for discovery of new potential biomarkers and design of drug targets in the future. However, challenges in generating miRNA-based therapeutics still remain, such as maintaining the stability of miRNAs for initial pre-clinical trials or animal model trials, and finding suitable carriers or delivery vehicles to enhance specificity and reduce toxicity when released inside the body [171]. Finally, in the era of precision medicine, where the focus is to find potential biomarkers and specific drug targets for treatment and detection of diseases, miRNAs could play a key role due to their proven stability, stress/disease specificity, and their conserved nature across phylogeny, allowing targeted action against specific subsets of gene transcripts.

## Data Availability

Data sharing not applicable.
